# Biomimetic Catechol-Incorporated Polyacrylonitrile Nanofiber Scaffolds for Tissue Engineering of Functional Salivary Glands

**DOI:** 10.34133/bmr.0226

**Published:** 2025-07-02

**Authors:** Seokjun Kwon, Ji Hyun Ryu, Junchul Kim, Hyun Ho Shin, Gehoon Chung, Ali Taghizadeh, Jung-Hwan Lee, Jongho Kim, Bon-Cheol Ku, Kyungpyo Park, Sang-woo Lee

**Affiliations:** ^1^ Department of Physiology, School of Dentistry and Dental Research Institute, Seoul National University, Seoul, Republic of Korea.; ^2^ Department of Biomedical Materials Science, Graduate School of JABA, Department of Carbon Convergence Engineering, Department of Chemical Engineering, Smart Convergence Materials Analysis Center, Wonkwang University, Iksan, Jeonbuk, Republic of Korea.; ^3^ Institute of Tissue Regeneration Engineering (ITREN), Dankook University, Cheonan, Chungcheongnam-do, Republic of Korea.; ^4^ Department of Textile System Engineering, Kyungpook National University, Daegu, Republic of Korea.; ^5^ Institute of Advanced Composite Materials, Korea Institute of Science and Technology (KIST), Wanju, Republic of Korea.; ^6^ Center for Nanoparticle Research, Institute for Basic Science (IBS), Seoul, Republic of Korea.

## Abstract

Replacing damaged salivary glands with in vitro-generated artificial glands offers a fundamental solution for salivary gland dysfunction. However, this approach remains challenging due to the gland's complex structure and cellular heterogeneity. Since natural organogenesis of salivary glands successfully orchestrates these complex processes, replicating the developmental niche in vitro is considered a promising solution. However, it consists of complex, branched structures formed by multiple factors; thus, recapitulation of these factors in vitro using a single type of biomaterial is difficult to achieve. Therefore, this study aims to design a scaffold capable of spontaneously mimicking salivary gland’s developmental niche. Herein, we demonstrate that catechol-incorporated polyacrylonitrile (PAN-C) nanofiber scaffold spontaneously transforms into biomimetic structures by adsorbing embryonic mesenchyme-derived extracellular matrix (ECM) and growth factors. Accumulated adsorption of ECM and growth factors on PAN-C nanofibers promoted the proliferation, morphogenesis, and functional differentiation of embryonic salivary gland (eSG) organoids in vitro. Transcriptome analysis revealed that the PAN-C nanofiber scaffold effectively reduced mechanical stress-induced gene expression while promoting proliferation and differentiation of salivary gland epithelial cells. In eSG organoids cultured on PAN-C nanofiber scaffolds, the proportion of functional acinar cells expressing apically localized aquaporin-5 was substantially higher than those cultured on polycarbonate membranes, a conventional culture material. Therefore, PAN-C nanofiber scaffolds provide an effective and economical method for generating functional eSG organoids in vitro.

## Introduction

The physiological functions of organs arise from the integrated activities of their specialized functional units, including acini in salivary glands, nephrons in kidneys, alveoli in lungs, lactating alveolar units in mammary glands, and hepatic lobules in the liver [1,2]. The development and maintenance of these organ-specific functional units depend critically on unique microenvironmental factors, such as appropriate viscoelastic properties, structural topology, and specific biological signals. Consequently, effective functional tissue engineering of complex organs necessitates culture platforms capable of accurately replicating and sustaining these specialized organ-specific microenvironments [3].

Salivary glands are sophisticated, multicellular exocrine organs composed of diverse cell types, including acinar, ductal, myoepithelial, vascular, and neural cells [3,4]. These cells collaborate to produce saliva, essential for maintaining both oral and systemic health. However, replicating the complex glandular morphology in vitro is a major challenge, as recreating the intercellular interactions and structural arrangement that enable saliva secretion is difficult. Current therapeutic options for conditions such as xerostomia provide only short-term relief, focusing on symptom management [4,5]. In contrast, recent developments in regenerative medicine suggest that transplanting engineered salivary gland organoids could provide a more sustainable solution [6]. However, producing functional organoids with the proper structural and functional characteristics remains challenging.

The complex architecture and heterogeneity of the salivary glands naturally arise through organogenesis, a process where cells organize into functional structures driven by precise biochemical and biomechanical cues [2,3,7]. Therefore, mimicking aspects of this developmental process in vitro is essential to achieve organoids with comparable structure and function to native glands. Recreating salivary gland organogenesis in vitro requires promoting the proliferation of epithelial cells, supporting the interactions between embryonic epithelial and mesenchymal cells for branching morphogenesis, and guiding the differentiation of progenitor cells into functional acinar cells that express aquaporin 5 (AQP5), a critical marker of salivary function [8].

**Fig. 1.**
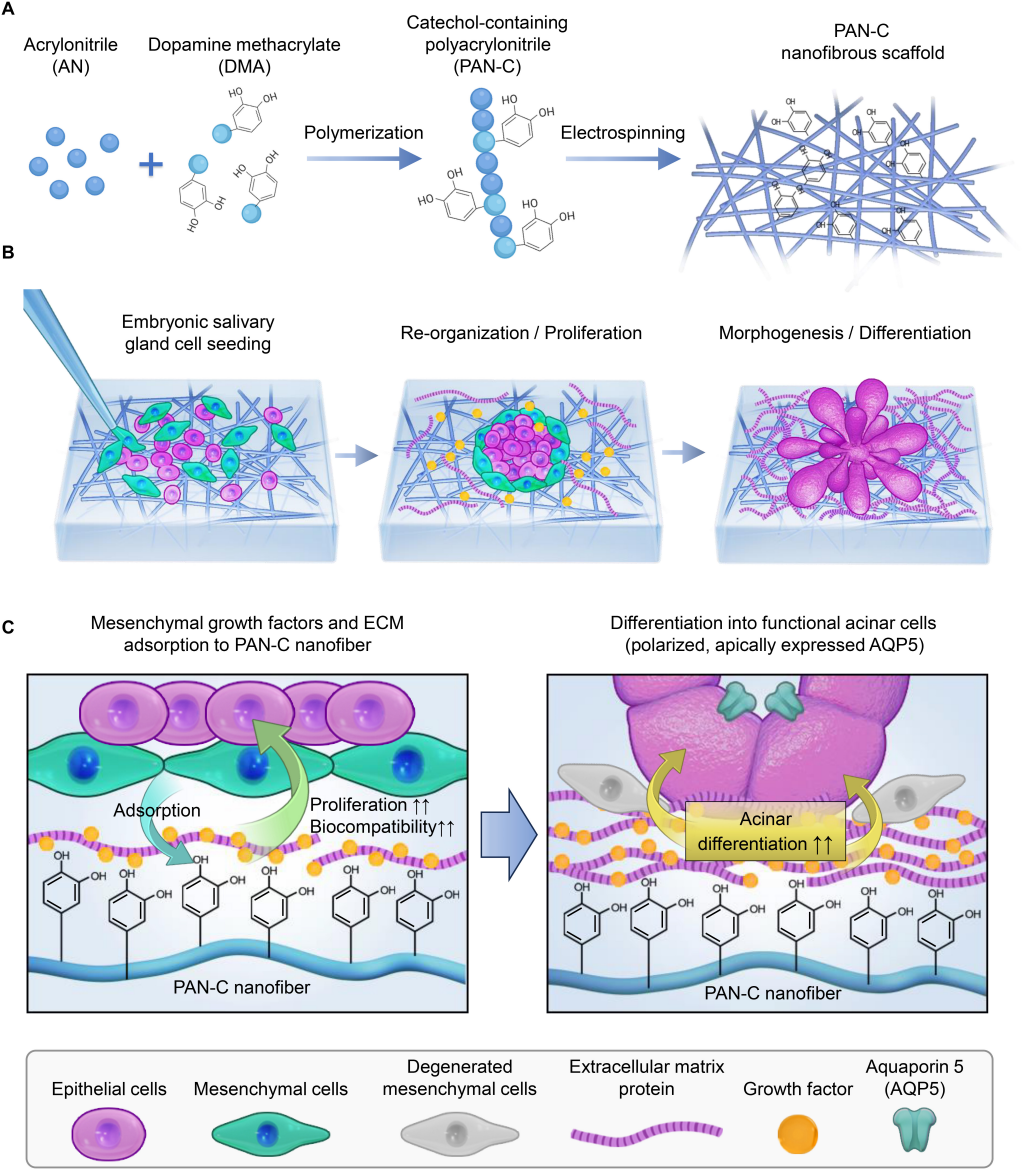
Schematic diagram of the catechol-incorporated polyacrylonitrile (PAN-C) nanofibrous embryonic salivary gland (eSG) organoids culture platform. (A) Synthesis of the PAN-C nanofibrous scaffold. (B) Establishment of salivary gland organoids on PAN-C using eSG cells. (C) Mechanism of enhanced proliferation and functional differentiation of eSG organoids: The adsorption of extracellular matrix proteins and growth factors secreted by embryonic mesenchymal cells onto PAN-C nanofibers creates a long-lasting niche that supports the proliferation and acinar differentiation of eSG organoids, even after the degeneration of mesenchymal cells.

This study introduces a biomimetic approach using polyacrylonitrile (PAN)-based nanofibrous scaffolds, specifically catechol-incorporated PAN (PAN-C), to address these challenges. PAN was selected due to its exceptional mechanical stability, chemical resilience, and capacity for nanofiber formation [9,10]. In particular, its nanofibrous structure closely mimics the topological characteristics of embryonic mesenchyme, which is essential for supporting epithelial morphogenesis and functional differentiation. However, the native PAN nanofibers are significantly stiffer than the soft, viscoelastic environment required for salivary gland organogenesis, and this rigidity can hinder cell proliferation, morphogenesis, and differentiation [4,11]. Additionally, conventional PAN lacks the bioactivity necessary for effective cell and growth factor interactions. To address these limitations, we incorporated catechol groups into PAN, leveraging their ability to bind various biomolecules to enhance the scaffold’s functionality and mitigate its stiffness-induced noxious effects (Fig. 1).

The resulting catechol-modified PAN-C nanofibers provide a dynamic surface that promotes the adsorption of essential extracellular matrix (ECM) components and growth factors, thus closely replicating the mesenchymal-like environment needed for salivary gland development (Fig. 1B and C). This modification successfully masks the high stiffness of the original PAN material, creating a more biocompatible niche that supports cell proliferation, branching morphogenesis, and acinar differentiation (Fig. 1B and C). By enabling the formation of functional embryonic salivary gland (eSG) organoids with structures and functions resembling native glands, this PAN-C scaffold approach offers a promising platform for regenerative approaches on various types of salivary gland dysfunctions.

## Materials and Methods

### Materials

Acrylonitrile (AN, Tokyo Chemical Industry Co. Ltd., Tokyo, Japan) was dried over CaH_2_. Dimethyl sulfoxide (DMSO), *N*,*N*-dimethylformamide (DMF), and 2,2′-azobisisobutyronitrile (AIBN) were obtained from Sigma-Aldrich (St. Louis, MO, USA). AIBN was freshly recrystallized using methanol before use.

### Synthesis of PAN-C

To synthesize PAN-C, the catechol monomer (CA) was initially prepared following a previously reported method [9,10]. PAN-C was synthesized via free radical polymerization of AN and CA. Briefly, AN, CA (97:3 molar fraction), and AIBN (5 × 10^−2^ M) were placed in a 3-neck double-layer flat-bottom reaction flask, followed by the addition of DMSO. After degassing through bubbling with N_2_ at room temperature for 2 h, the polymerization reaction was conducted at 60 °C under N_2_ condition for 16 h. After the reaction, the products were cooled to room temperature and poured into excess distilled water with vigorous stirring. The solid was filtered, washed thrice with distilled water, and dried at 60 °C under vacuum for 16 h until a constant weight was reached. The ratio of AN and CA units after polymerization was determined to be 96:4, measured using ^1^H-NMR spectrum (Agilent 600 MHz Premium COMPACT). The ^1^H-NMR spectra of PAN-C were measured in DMSO-d6 with tetramethylsilane (TMS, 0.03 vol%) as an internal standard.

### Preparation and characterizations of PAN-C nanofibrous membranes

PAN-C nanofibrous membranes were prepared using an electrospinning/spray system (ESR100D, NanoNC, Seoul, Republic of Korea). Briefly, PAN-C (10 wt%) was dissolved in DMF at room temperature until fully dissolved. The solution was then electrospun through a 21-gauge needle at a voltage of 20 kV. The flow rate was set at 80 μl/min, and the distance between the needle and the aluminum foil-wrapped drum collector was 18 cm. For control, an unmodified PAN nanofibrous membrane was prepared using the same method as described above.

For morphological analysis of PAN-C nanofibrous membranes, scanning electron microscopic (SEM) images were captured at an acceleration voltage of 5 kV. The PAN and PAN-C nanofibrous membranes were placed on the SEM holder using carbon tapes, followed by platinum deposition on the membranes.

The mechanical properties of the PAN and PAN-C nanofibers were measured using a 2-parallel plate compressive system (CellScale, MicroTester G2). The nanofibers (*n* = 3 for each composition) were placed on a fixed flat stage, and a constant force was applied using a stainless-steel platen attached to a tungsten microbeam. Displacement of the compression platen was tracked using a camera, while the nanofibers were compressed to a final strain of 20% of their diameter for 20 s. Force–displacement curves were automatically recorded by MicroTester software. The Young's modulus values of PAN and PAN-C were calculated as the force exerted on each sample under tension divided over the product of extensional strain and cross-sectional area.

Water contact angles (WCAs) of PAN and PAN-C nanofibrous membranes were measured using a Contact Angle Analyzer (PHX300 + Tilt, SEO, Suwon, Republic of Korea). The PAN and PAN-C films and nanofibrous membranes were placed on the plate, and contact angles of the water drop on the membranes were measured using video recording. In addition, PAN and PAN-C films were incubated in a pH 7.4 phosphate-buffered saline (PBS) solution containing 10 mM albumin for 4 h. Both films were vigorously washed with PBS and double-distilled water (DDW) at least 3 times and then dried. Subsequently, the WCAs were measured. Commercially available polycarbonate (PC) membranes, with/without albumin treatments, served as controls.

To evaluate the long-term structural stability of PAN and PAN-C nanofiber scaffolds, each membrane was immersed in Dulbecco’s Modified Eagle Medium (DMEM; Gibco) and incubated for 10 days at 37 °C in a humidified incubator with 5% CO_2_. After incubation, the samples were rinsed with distilled water, air-dried, and mounted for SEM analysis. The surface morphology of the nanofibers before and after incubation was imaged using SEM to assess morphological integrity and fiber preservation.

### Mesenchymal factor deposition assay

The PC membrane, PAN, and PAN-C were cut into 6-mm-diameter circles using a disposable 6.0-mm biopsy punch (Kai Medical, BP-60F). They were then treated with collagen type I–fluorescein isothiocyanate (FITC) conjugate (Sigma-Aldrich, C4361), laminin (Corning, 356231), heparin–fluorescein conjugate (Invitrogen, H7482), or chondroitin sulfate fluorescein (CD Bioparticles, CDHA104) at a concentration of 200 μg/ml. Treatment involved shaking at 120 rpm for 4 h at room temperature. To detect the laminin fluorescence signal, laminin Polyclonal Antibody-DyLight 488 (Invitrogen, PA5-22901) was further treated for 8 h at 4 °C. Each membrane and nanofiber was washed 4 times with PBS and mounted on a glass slide using a mounting medium (Fluoro-gel; PST proSciTech, IM030). Fluorescence images were captured using a confocal laser-scanning microscope (Carl Zeiss, LSM980).

### In vitro embryonic submandibular glands culture

The embryonic submandibular glands (eSMGs) extracted from ICR (Institute of Cancer Research) mouse (DooYeol Biotech, Korea) fetus at embryonic day 13.5 were cultured on PC membrane (Whatman, 110405), PAN, or PAN-C nanofibers following previously established protocols [12,13]. Briefly, eSMGs were cultured in DMEM/F12 1:1 media (Gibco, 21041-025) supplemented with 50 μg/ml transferrin (Sigma-Aldrich, T8158), 150 μg/ml ascorbic acid (Sigma-Aldrich, A5960), and 1% antibiotic–antimycotic (Gibco, 15240062) on substrates with air–media interface. The culture was incubated at 37 °C with 5% CO_2_ for 48 h. The animal experiment protocol used in this study was approved by the Seoul National University Institutional Animal Care and Use Committee.

### Immunofluorescence staining and imaging

The eSMGs or eSG gland organoids cultured for 48 h were fixed with 4% paraformaldehyde for 18 min and then washed with PBS at room temperature. Subsequently, the eSMGs or eSG organoids were blocked and permeabilized with PBS containing 0.1% Triton X-100 (PBSX), 10% normal donkey serum (NDS; Sigma-Aldrich, D9663), and 1% Mouse on Mouse blocking reagent (VectaShield Laboratory, BMK-2213-1) for 3 h at room temperature. After blocking and permeabilization, eSMGs or eSG organoids were incubated overnight at 4 °C with primary antibodies (1:100 to 200) diluted in PBSX containing 3% NDS. The primary antibodies used for immunofluorescence staining are as follows: oat polyclonal anti-c-kit antibody (R&D Systems, AF1356), beta-III tubulin antibody (TUJ1; R&D Systems, MAB1195), Ki67 monoclonal antibody (Invitrogen, 14-5698-82), and anti-aquaporin 5 antibody (Alomone Labs, AQP-005). After incubation, the primary antibody solutions were washed 4 times with PBS containing 0.5% Tween 20 (PBST) at room temperature, with each wash lasting 10 min. After washing, eSMGs or eSG organoids were incubated overnight at 4 °C with secondary antibodies (1:250) or peanut lectin agglutinin conjugated with FITC (diluted 1:20) in PBSX containing 3% NDS and 4′,6-diamidino-2-phenylindole (DAPI; 1:1,000). The secondary antibodies used for immunofluorescence staining are as follows: donkey anti-goat IgG (H+L) Alexa Fluor 488 conjugate (Abcam, ab150129), Alexa Fluor 488 donkey anti-goat IgG (H+L) (Jackson ImmunoResearch, 705-545-003), Alexa Fluor 594 donkey anti-mouse IgG (H+L) (Invitrogen, A-21203), Alexa Fluor 647 donkey anti-rat IgG (H+L) (abcam, ab150155), and Alexa Fluor 488 donkey anti-rabbit IgG (H+L) (Invitrogen, A-21206). The secondary antibody solutions were washed 4 times with PBST (10 min per wash) at room temperature. After washing, eSMG or eSG organoids were mounted on glass slides using Fluoro-gel (PST proSciTech, IM030). Fluorescence-stained images of eSMGs or eSG organoids were captured using a confocal laser-scanning microscope (Carl Zeiss, LSM980). Bright-field images of cultured eSMGs or eSG organoids were acquired using an inverted microscope (Nikon, DS-Ri2/Nikon Ti) to observe morphological changes over 0, 24, and 48 h.

### Substrate stiffness-modulated culture of eSMGs

To investigate the effect of substrate stiffness on eSMG development, agar hydrogels of varying concentrations (0.5%, 1%, 2%, and 4% w/v) were prepared by dissolving low-melting agar in PBS and casting into 6-well plates. The Young’s modulus of each hydrogel was measured using a rheometer (TA instruments, Discovery Hybrid Rheometer) under frequency-sweep mode (0.1 to 10 Hz) with an 8-mm parallel plate. The representative Young’s modulus was measured at 1 Hz.

Embryonic day 13.5 eSMGs were isolated and cultured on the surface of each hydrogel at the air–media interface for 48 h, following the same conditions as described for standard PC membrane cultures. Bud number was manually quantified from bright-field images. In addition, immunofluorescence staining was performed with Ki67 and DAPI to evaluate epithelial proliferation across hydrogel conditions.

### eSMG RNA extraction and sequencing data analysis

RNA acquisition from cultured or freshly extracted eSMGs and library construction for sequencing were performed as previously described [14]. Briefly, total RNA from up to 10 eSMGs per sample was isolated following the protocol provided on the Direct-zol RNA MiniPrep kit (Zymo Research, R2050; Tustin, CA, USA). Subsequent library preparation for sequencing was performed by Ebiogen, Inc. (Seoul, Korea), utilizing the QuantSeq 3′ mRNA-Seq Library Prep Kit (Lexogen, Greenland, NH, USA). Sequencing was conducted on an Illumina NextSeq 500 platform (Illumina, San Diego, CA, USA), generating 50 base pair single-end reads. Raw read counts were normalized using the edgeR package in R, employing quantile normalization. Fold changes were calculated from the normalized data, and *P* values were determined using a 2-sample *t* test. Ingenuity pathway analysis (IPA, Qiagen Inc., version 94302991) was used to interpret the biological significance of the differentially expressed genes (DEGs) identified in our study. DEGs with a fold change greater than 1.5 and *P* value < 0.05 were analyzed using IPA to investigate related canonical pathways and upstream regulators.

### eSG organoid generation and culture

eSMGs (35 to 50) isolated from ICR mouse fetus at embryonic day 15 were immersed in RPMI media (Cytiva, SH30255.01) supplemented with 1 mg/ml type I and II collagenase (Roche, 05401020001) and incubated at 37 °C for 20 min, with rotation using the MACSmix Tube Rotator (Miltenyi Biotec, 130-090-753). eSMG tissues were mechanically dissociated using the pre-programmed hTumor 1, 2, and 3 modes of the gentleMACS Dissociator (Miltenyi Biotec, 130-093-235). The dissociated eSMG was subsequently filtered through 70-μm MACS SmartStrainers (Miltenyi Biotec, 130-098-462). Following centrifugation, the resulting supernatant was discarded, and 50 μl of RPMI was added. The dissociated eSMG was seeded onto either PC membrane or PAN-C using 5 μl and then incubated for 48 h.

### In vivo biocompatibility of PAN-C

To evaluate the long-term biocompatibility of PAN-C scaffolds, ultraviolet-sterilized nanofiber membranes (6.0 mm diameter) were implanted to submandibular glands (SMGs) in adult female ICR mice (10 weeks old). Mice were anesthetized, and a small incision was made in the ventral neck region to expose the SMG. PAN-C membranes were placed on the glands, and the incision was sutured. Body weight was monitored for 7 days. At the end of the observation period, mice were sacrificed and SMG tissues were collected for histological analysis using hematoxylin and eosin (H&E) staining.

### Data collection and statistical analysis

The number of buds on eSMGs was manually counted from the bright-field images. Bud and eSG organoid sizes were measured using ImageJ Software. The proliferation of eSMG (Ki67-positive and DAPI-positive) and the parasympathetic innervation (TUJ1-positive and PLA-positive) was quantified using Zen 2010 Blue software (Carl Zeiss). To evaluate ductal lumen formation in eSMG, the PLA-positive intensity along the line across the duct was calculated using Zen 2010 Blue software. Statistical analysis and significance were calculated using one-way analysis of variance (ANOVA) with Tukey’s multiple comparison tests in Prism 9.1.0 (GraphPad Software, Inc.). Significance was set to *P < 0.05, **P < 0.01, ***P < 0.005, ****P < 0.001.

## Results

### Preparation and characterization of PAN and PAN-C

SEM images revealed that the mesenchyme of the eSMG consisted of densely packed fibrous structures, suggesting that a fibrous scaffold is suitable for in vitro organotypic culture of the salivary gland (Fig. S1). To mimic the structure of eSMG mesenchyme, PAN and PAN-C were used to prepare the fiber structures. PAN is one of the most commonly used materials for preparing nanofibers through electrospinning [15,16]. PAN nanofibers demonstrate excellent mechanical properties, chemical stability, and electrospinnability without obvious toxicity [15]. In addition, catechol groups can bind to various biomolecules, including proteins associated with the ECM [17,18]. Thus, we hypothesized that PAN-C nanofibers effectively mimic the topological and biological properties of embryonic mesenchyme.

**Fig. 2.**
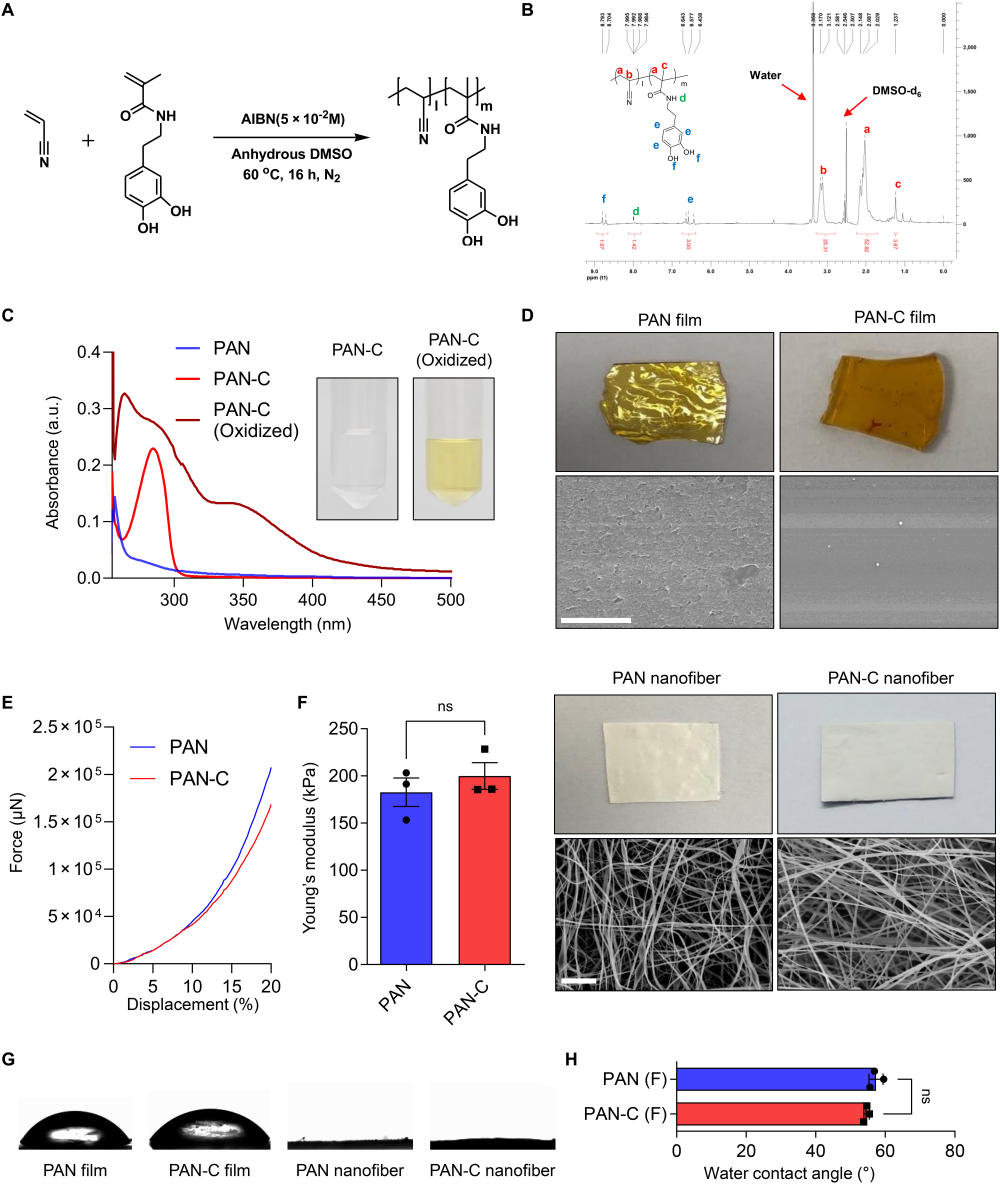
Physicochemical characterization of polyacrylonitrile (PAN) and PAN-C. (A) Radical polymerizations of acrylonitrile and dopamine methacrylate monomers to synthesize PAN-C. (B) ^1^H-NMR spectra of PAN-C. (C) UV–Vis spectrum graphs of PAN, PAN-C, and oxidized PAN-C. (D) Bright-field and SEM images of films (top) and nanofibers (bottom) of PAN and PAN-C. Scale bar = 20 μm. (E) Force–displacement curve of PAN and PAN-C. (F) Young’s modulus of PAN and PAN-C measured from (E) (*n* = 3). (G and H) Water contact angle measurement of PAN film, PAN-C film, PAN nanofiber, and PAN-C nanofiber (*n* = 3) (ns, not statistically significant).

PAN-C was synthesized through free radical polymerization of AN and CA using AIBN (Fig. 2A). The catechol groups in PAN-C were confirmed with ^1^H-NMR (Fig. 2B). The catechol proteins (-CH3, 3H) were assigned to the range of 6.5 to 7.0 parts per million, with the absorption of peak labeled with blue alphabet symbols. The calculated ratio of AN/CA units of PAN-C was 96:4. In addition, the catechol oxidation of PAN-C was confirmed using ultraviolet–visible (UV–Vis) spectroscopy (Fig. 2C). The absorbance of PAN-C at 280 nm wavelength in DMF co-solvents, as well as its adsorption around 350 nm, was observed, showing an overall upshift in the spectrum. As previously reported, the oxidation of catechol groups to catecholquinone groups produces shoulders around 350 nm [19]. These findings suggest that the catechol groups of PAN-C had the potential to react with various biomacromolecules. PAN and PAN-C films, as well as PAN and PAN-C nanofibrous membranes, were prepared for material characterizations (Fig. 2D). PAN and PAN-C films were prepared using solution casting methods (Fig. 2D, top), while PAN and PAN-C nanofibrous membranes were prepared using electrospinning methods. The nonwoven structures of PAN and PAN-C nanofibrous membranes were confirmed by SEM images (Fig. 2D, bottom). The fiber diameters of PAN and PAN-C nanofibers showed slight differences; however, no statistical significance was observed (Fig. 2D, third and fourth panels). Mechanical properties such as substrate stiffness are known to significantly affect the growth, morphogenesis, and differentiation of salivary glands [11]. To measure the stiffness or Young’s modulus of PAN and PAN-C nanofiber scaffold, a force–displacement curve was obtained. The mean values of Young’s modulus of PAN and PAN-C were 182.61 ± 12.34 kPa and 199.93 ± 11.62 kPa, respectively (*P* > 0.5), indicating no significant difference in substrate stiffness (Fig. 2E and F). WCAs were measured for PAN and PAN-C to confirm their wettability. The WCAs of PAN and PAN-C films were 57.0 ± 0.7° and 54.5 ± 0.7°, respectively (*P* > 0.5), indicating that the presence of catechol did not alter the hydrophilicity of PAN (Fig. 1G and H). In nanofiber scaffold form, PAN and PAN-C completely absorbed a water droplet within 10 s, ensuring homogeneous distribution of culture media in an air–media interface culture system. Next, we evaluated the structural integrity of PAN and PAN-C nanofibers under long-term culture conditions. Scaffolds were immersed in DMEM and incubated for 10 days at standard cell culture conditions, followed by morphological assessment using SEM. As shown in Fig. S2, neither PAN nor PAN-C nanofibers exhibited significant morphological alterations after incubation. Additionally, no notable morphological differences were observed between PAN and PAN-C scaffolds, confirming their long-term structural stability.

Overall, the presence of catechol in PAN did not significantly alter the physicochemical properties of the PAN film or nanofiber.

### Enhanced biocompatibility of PAN-C nanofiber scaffold compared to PAN

**Fig. 3.**
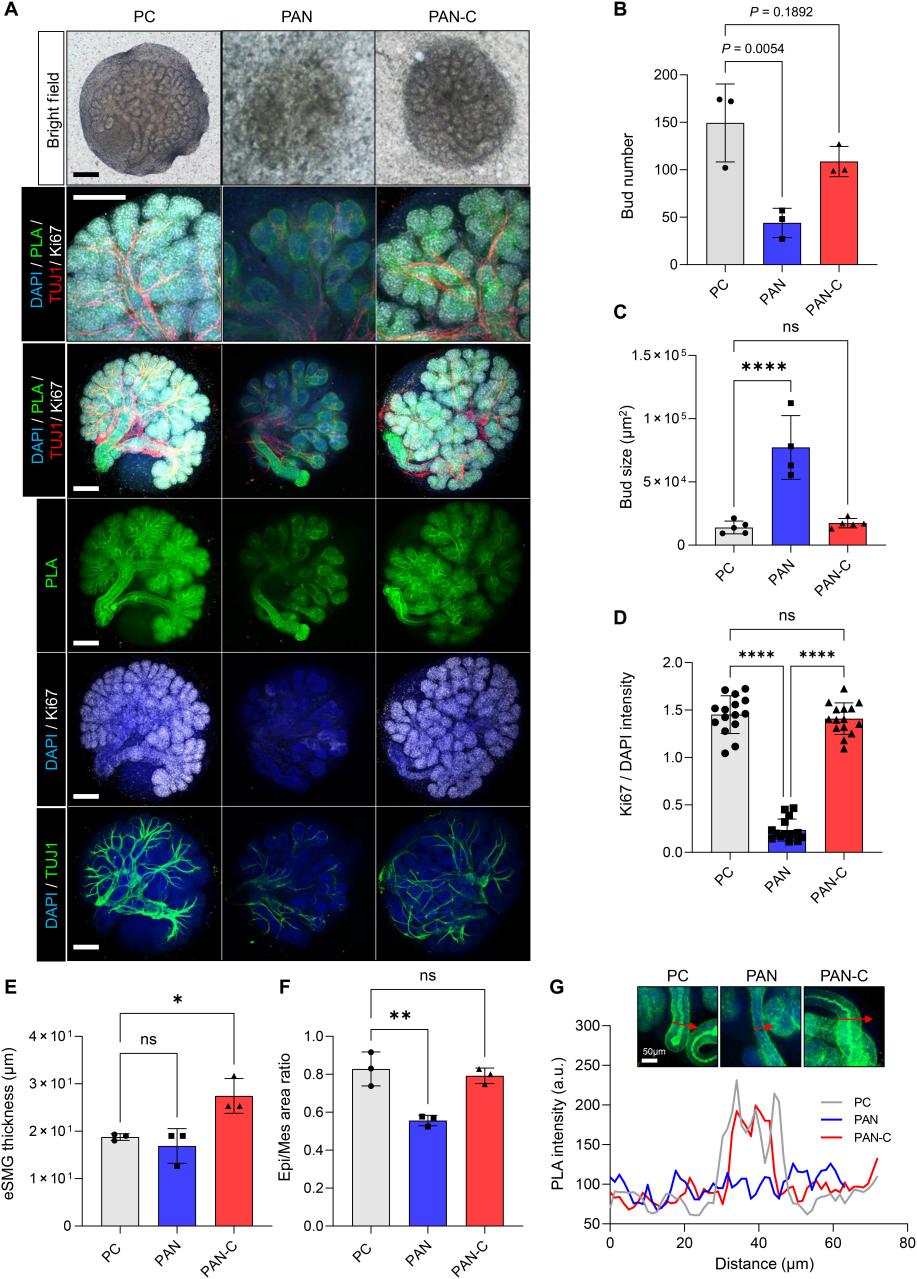
Branching morphogenesis of embryonic submandibular glands (eSMGs) cultured on polycarbonate membrane (PC), PAN nanofiber, and PAN-C nanofiber. (A) Bright-field images (top) and immunofluorescence images of eSMGs stained with DAPI (blue), Ki67 (white), PLA (green), and TUJ1 (red). Scale bar = 200 μm. (B to F) Quantification of bud number, bud size, Ki67 fluorescence intensity over DAPI, the thickness of eSMGs, and area ratio of epithelium over mesenchyme (*n* = 3 to 30). (G) Confocal images and cross-sectional quantification of PLA fluorescence intensity for lumen formation in duct. Scale bar = 50 μm. The red arrows indicate vertical cross-sections of each duct. Statistical significance is set as *P < 0.05, **P < 0.01, ****P < 0.0001; ns, not statistically significant.

**Fig. 4.**
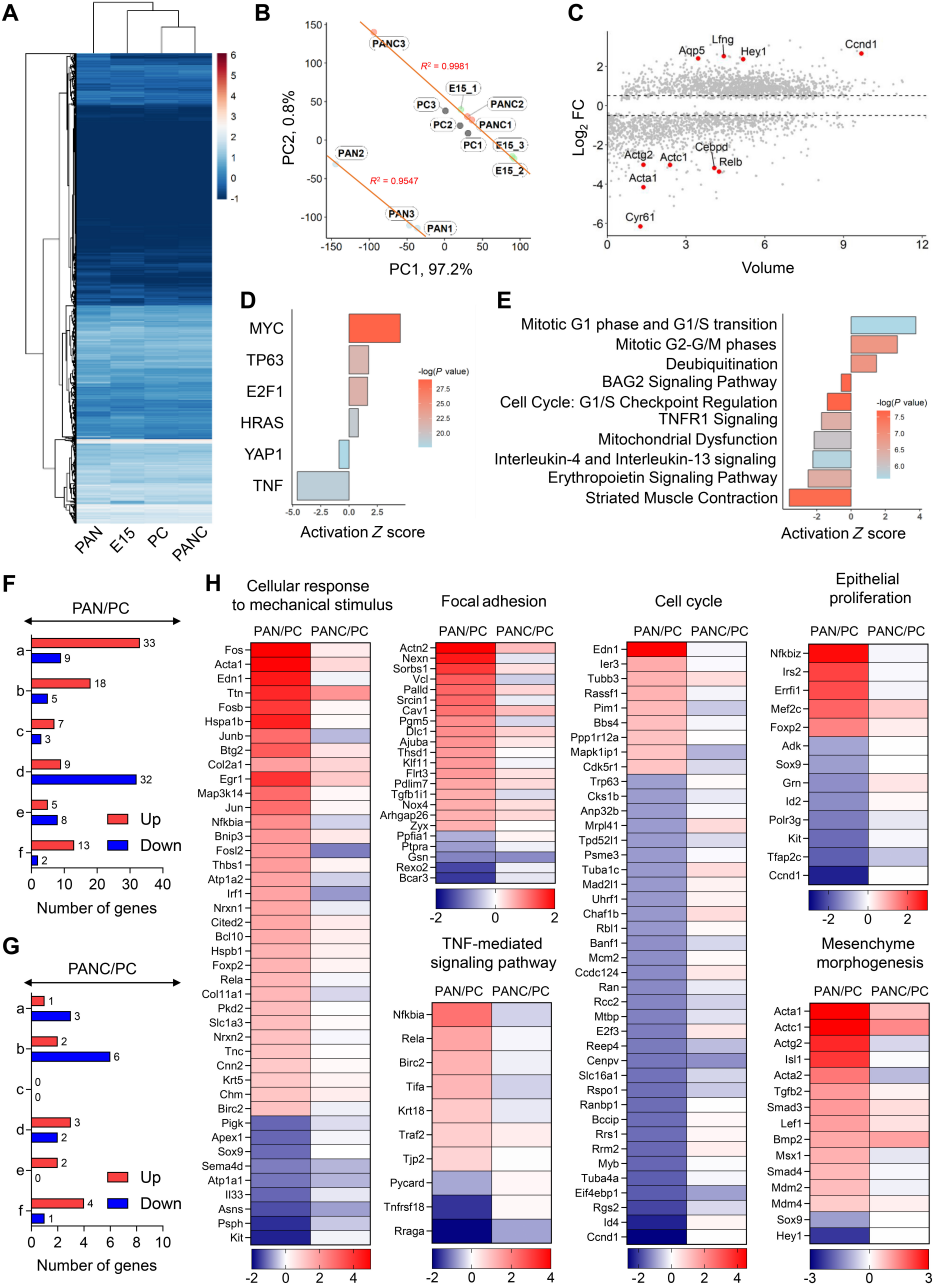
Transcriptome analysis of eSMGs cultured on PC, PAN nanofiber, and PAN-C nanofiber. (A) Heatmap showing hierarchical clustering of gene expression profiles across PC, PAN, PAN-C, and embryonic day 15 groups. (B) Principal component analysis of the expression profiles, depicting the variance captured by the first and second principal components. (C) Volcano plot of fold changes in gene expression between PAN-C over PAN groups. (D) Upstream regulators analysis conducted using ingenuity pathway analysis (IPA), highlighting key regulatory molecules. (E) Canonical pathways analysis results from IPA, showing significantly activated or inhibited pathways. (F and G) Number of genes significantly up-regulated and down-regulated (>1.5-fold change with volume > 4) in each gene ontology (GO); eSMGs cultured on PAN over those cultured on PC (F) and eSMGs cultured on PAN-C over those cultured on PC (G). (a) Cellular response to mechanical stimulus (GO:0071260), (b) focal adhesion (GO:0005925), (c) TNF-mediated signaling pathway (GO:0010803), (d) cell cycle (GO:0007049), (e) epithelial proliferation (GO:0050673), and (f) mesenchyme morphogenesis (GO:0072132). (H) Heatmap of GO-categorized genes with significant expressional changes (>1.5 fold change with volume > 4) in eSMGs cultured on PAN over those cultured on PC. Expressional fold changes of the selected genes in eSMGs cultured on PAN-C over those cultured on PC are analyzed in parallel.

An ideal biomaterial supporting the in vitro generation of artificial salivary glands should promote stable proliferation, branching morphogenesis, functional neural innervation, and structural maturation of the salivary gland. Therefore, the eSMG ex vivo organotypic culture model has been widely utilized to evaluate these parameters [2,13]. To assess the sustainability and biocompatibility of PAN or PAN-C in in vitro organ-level culture of salivary glands, eSMGs isolated from embryonic day 13.5 were placed on PC, PAN nanofiber, and PAN-C nanofiber and cultured using an air–media interface. As eSMG culture on PC is a widely adopted conventional method, they serve as the control group. We examined parameters including bud number, bud size, eSMG thickness, epithelial/mesenchyme ratio, expression of epithelial proliferation marker (Ki67), and degree of tubulogenesis and parasympathetic innervation. The eSMGs cultured on PAN exhibited significant retardation of branching morphogenesis (70% reduction in bud number and a 5-fold increase in unbranched end bud), along with a lack of Ki67-positive proliferative cells (84% reduction in Ki67/DAPI intensity) and parasympathetic innervation, compared to those of eSMGs cultured on PC (Fig. 3A to D). However, for eSMGs cultured on PAN-C, all the parameters mentioned above were comparable to those of eSMGs cultured on PC, with no statistically significant differences observed (Fig. 3A to D). The average thickness of eSMGs cultured on PAN-C was higher than that of eSMGs cultured on PC, suggesting that the nanofibrous structure provides a 2.5-dimensional culture condition (Fig. 3E). The epithelial/mesenchyme ratio is an indicator of salivary gland maturation, as the mesenchyme diminishes while the epithelium radically increases in volume during the branching morphogenesis process [2]. eSMGs cultured on PAN exhibited a significantly lower epithelium/mesenchyme ratio, indicating delayed maturation, while those cultured on PAN-C exhibited a comparable epithelium/mesenchyme ratio to that of the PC group (Fig. 3F). Ductal tubulogenesis is another developmental indicator of salivary gland maturation. In the early stages of development, the salivary gland duct is closed, but by embryonic day 15, it begins forming a lumen through a process called tubulogenesis [20], which can be visualized with PLA-FITC. eSMGs cultured on PAN exhibited no observable luminal structure in the excretory duct. In contrast, those cultured on PAN-C exhibited approximately 20-μm-diameter lumens, comparable to that of eSMGs cultured on PC (Fig. 4G). These findings indicate that while PAN nanofiber alone is not suitable for salivary gland culture, the presence of catechol can markedly enhance its biocompatibility.

To test if the intrinsic cytotoxicity of PAN contributes to impaired morphogenesis, we separately cocultured eSMGs with PAN nanofiber scaffolds, thereby avoiding direct contact between them. Under these conditions, eSMGs exhibited normal branching morphogenesis and proliferation comparable to those cultured on PC membrane. These results indicate that the PAN-induced retardation of eSMG branching morphogenesis was not due to the cytotoxic substances released from PAN (Fig. S3).

Next, we further evaluated long-term biocompatibility of PAN-C nanofiber scaffolds in vivo. PAN-C nanofiber scaffolds were surgically implanted onto the SMGs of adult mice and maintained in situ for 7 days. Body weight changes between PAN-C implanted mice and control groups remained comparable throughout the implantation period, suggesting no systemic toxicity (Fig. S4A). Additionally, histological examination of salivary gland tissues using H&E staining showed no signs of necrosis, immune cell infiltration, or fibrosis (Fig. S4B). These results confirm that PAN-C scaffolds do not exhibit toxicity over an extended period, highlighting their suitability for future implantable and regenerative applications.

### PAN-C mitigates growth inhibition induced by substrate stiffness and mechanical stress

To elucidate the molecular mechanisms underlying catechol-dependent changes in the biocompatibility of PAN, we performed mRNA sequencing on embryonic day 13 eSMGs. These eSMGs were cultured on PC, PAN, and PAN-C for 48 h. Additionally, freshly isolated embryonic day 15 eSMGs were included as a reference group. Hierarchical clustering analysis of the expression heatmap revealed that the gene expression profile of the PAN-C group was more closely related to the PC group than to the PAN group, indicating that eSMGs cultured on PAN-C is more closely mimicking the natural developmental process than those cultured on PC, the gold standard material (Fig. 4A). We then conducted a principal component analysis on the entire gene set, where the first principal component (PC1) accounted for 97.2% of the total variance and the second principal component (PC2) explained 0.8% (Fig. 4B). The samples from the PAN group were positioned diagonally opposite to the PC, PAN-C, and embryonic day 15 groups. In the PAN group, 3 samples showed a high correlation coefficient of 0.99, while the other samples exhibited a correlation coefficient of 0.95. This suggests that the influence of PAN on eSMGs is significantly reduced in PAN-C samples, aligning more closely with the PC and embryonic day 15 groups. Subsequently, we identified genes significantly regulated in the PAN-C over PAN comparison (Fig. 4C). In the PAN-C group, key up-regulated genes including Ccnd1, Lfng, Aqp5, and Hey1, known for their roles in cellular growth and differentiation, highlight the enhanced biocompatibility of the PAN-C platform. Ccnd1 supports cell cycle progression, while Lfng and Hey1 activate the Notch signaling pathway, promoting developmental processes and cell fate determination [21]. The abundant expression of Aqp5, a marker indicating terminal differentiation of acinar cells, indicates the restored functional capability of eSMGs in the PAN-C platform [8]. In contrast, significantly down-regulated genes included Actg2, Actc1, Cebpd, Relb, Acta1, and Cyr61. These genes are involved in muscular function, inflammation, and cellular stress responses. The down-regulation of Actg2 and Actc1 suggests reduced muscular stress, while low expression of Cebpd and Relb indicates a lower inflammatory state. Additionally, the down-regulation of Cyr61, a downstream target gene of Yes-associated protein (YAP), suggests reduced mechanical strain [22–24].

To further elucidate the differences between the PAN and PAN-C groups, we utilized IPA. We analyzed genes with a fold change > 1.5 and *P* value < 0.05 in the differential expression of PAN-C compared to that of PAN. The upstream regulators analysis identified MYC, TP63, E2F1, and HRAS as primary activating regulators, with YAP1 and TNF highlighted as the main inhibiting regulators (Fig. 4D). These findings indicate that eSMGs cultured on PAN-C exhibit increased proliferation without activating stiffness-sensing YAP and related inflammatory signaling pathways than those cultured on PAN. The canonical pathways analysis revealed activations of pathways involved in cell cycle progression, particularly the mitotic G1 phase and G1/S transition, as well as the mitotic G2/M phase. Conversely, pathways associated with immune response, such as tumor necrosis factor receptor 1 (TNFR1) signaling and interleukin-4 and interleukin-13 signaling, along with pathways related to cellular dysfunction such as mitochondrial dysfunction, were inhibited (Fig. 4E). Additionally, the significant reduction in the activity of the striated muscle contraction pathway suggests that the PAN-C platform mitigates the mechanical stress exacerbated by PAN. During salivary gland morphogenesis, YAP/TAZ activation has been proposed to be associated with the maintenance of epithelial stem/progenitor cells [25]. Therefore, we further dissected the transcriptomes based on the following gene ontologies expected to sequentially interact from mechano-sensing to morphogenesis of salivary glands: “Cellular Response to Mechanical Stimulus” (GO:0071260), “Focal Adhesion” (GO:0005925), “TNF-mediated cell signaling pathway” (GO:0010803), “Cell cycle” (GO:0007049), “Epithelial Proliferation” (GO:0050673), and “Mesenchyme Morphogenesis” (GO:0072132). Overall, a large number of genes associated with “Cellular Response to Mechanical Stimulus” (labeled as a in Fig. 4F and G), “Focal Adhesion” (labeled as b in Fig. 4F and G), “TNF-mediated Signaling Pathway” (labeled as c in Fig. 4F and G), and “Mesenchyme Morphogenesis” (labeled as f in Fig. 4F and G) were significantly up-regulated, while those related to “Cell Cycle” and “Epithelial Proliferation” were significantly down-regulated in eSMGs cultured on PAN compared to those cultured on PC (Fig. 4F to H). In contrast, eSMGs cultured on PAN-C showed minimal alteration in gene expressions within these gene ontologies (Fig. 4F to H). SOX9 [26] and Kit [2], expressed in key salivary gland stem/progenitor cell populations for regeneration and organogenesis, were significantly down-regulated in eSMGs cultured on PAN, emphasizing the pivotal role of the mechanical properties of the scaffold in salivary gland tissue engineering (Fig. 4H, “Epithelial Proliferation”).

To further confirm whether substrate stiffness contributes to the inhibition of eSMG proliferation and branching morphogenesis, we cultured eSMGs on agarose hydrogels with varying concentrations to simulate different stiffness levels. The measured Young’s moduli of 0.5%, 1%, 2%, and 4% agarose gels were 4.15, 23.21, 115.67, and 162.34 kPa, respectively (Fig. S5A). When cultured on these substrates, eSMGs exhibited decreased bud numbers with increasing hydrogel stiffness (Fig. S5B and C). Notably, eSMGs cultured on the softest hydrogel (4.15 kPa) displayed a bud number comparable to those cultured on standard PC membranes (Fig. S5B and C), suggesting a threshold stiffness between approximately 4 and 23 kPa for significant inhibition of branching morphogenesis. Furthermore, eSMGs cultured on 4% agarose hydrogel (162.34 kPa), exhibiting stiffness similar to PAN and PAN-C nanofiber scaffolds (182.61 ± 12.34 and 199.93 ± 11.62 kPa, respectively), demonstrated comparable degeneration and a reduction in Ki67-positive proliferating cells, as observed on PAN scaffolds (Fig. S5B to E). These findings collectively indicate that the high stiffness of PAN nanofiber scaffolds is a primary factor responsible for the observed growth inhibition.

### PAN-C is capable of effectively adsorbing critical ECM proteins and growth factors

**Fig. 5.**
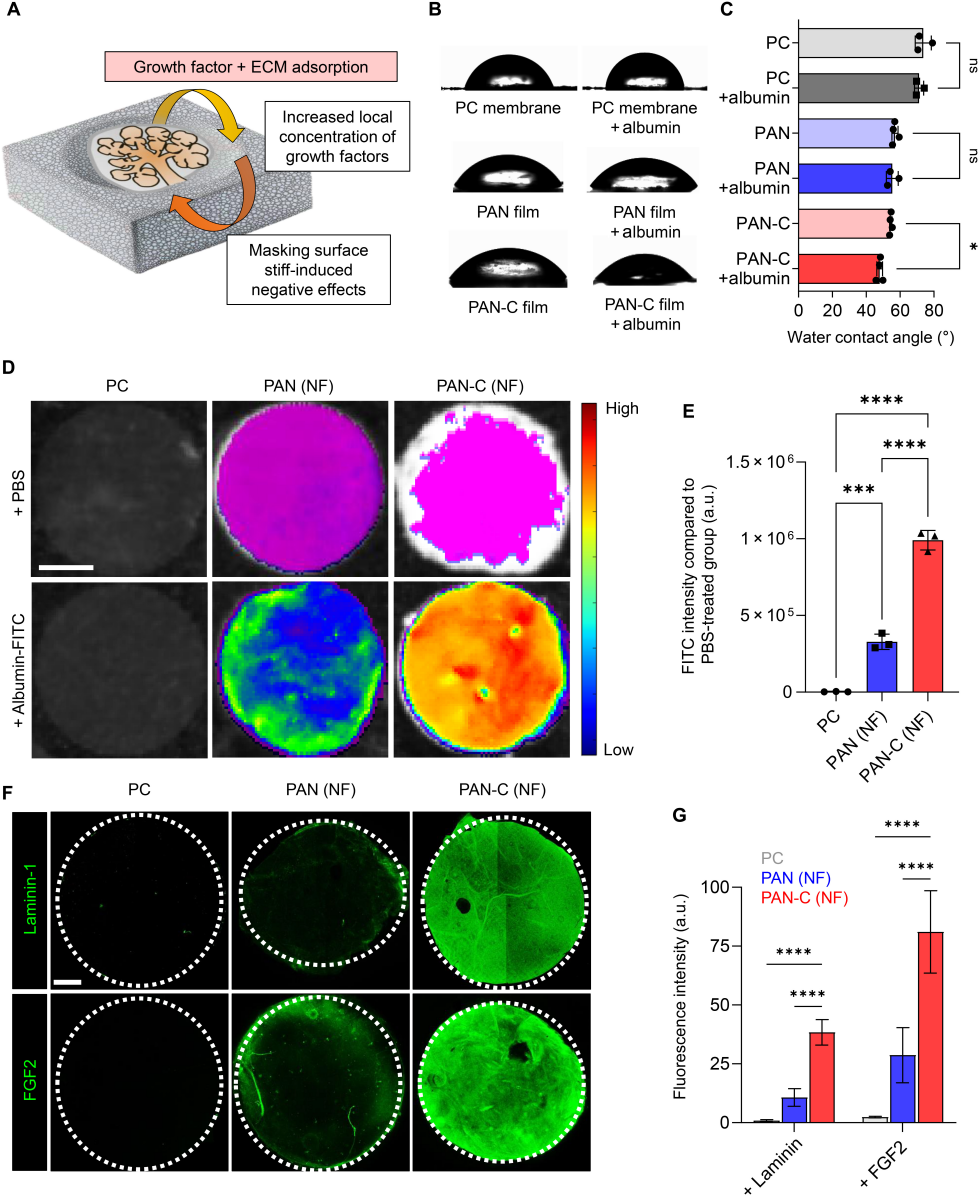
Protein deposition assay on PC, PAN nanofiber, and PAN-C nanofiber. (A) Schematic illustration depicting the adsorption of mesenchymal factors on PAN-C. (B and C) Water contact angle measurement of PC, PAN, and PAN-C with and without albumin (*n* = 3 to 5). (D) Merged bright-field image and relative fluorescence heatmap showing PC, PAN, and PAN-C treated with PBS or albumin-FITC. Scale bar = 2 mm. (E) The fluorescence intensities of the albumin-treated group were divided by the PBS-treated group (*n* = 3). (F) Confocal images of PC, PAN, and PAN-C treated and stained with laminin-1 or fibroblast growth factor 2 (FGF-2). The white dotted circles indicate the boundary of each circular membrane. Scale bar = 1 mm. (G) The fluorescence intensities of laminin-1 and FGF-2 were measured (*n* = 3). Statistical significance is set as *P < 0.05, ***P < 0.001, ****P < 0.0001; ns, not statistically significant.

Having found that the high surface stiffness of PAN was crucial in retarding salivary gland branching morphogenesis and maturation, we hypothesized that the enhanced biocompatibility of PAN-C resulted from ECM and growth factor deposition on catechol moieties, thereby masking the negative effects of high stiffness. Catechol moieties are known for their ability to readily immobilize organic substances such as proteins, polymers, peptides, and oligonucleotides [17,18]. As mentioned earlier, the mesenchyme of eSMGs produces various ECM components, including laminin-111, collagen 4, and glycosaminoglycan, along with growth factors such as fibroblast growth factor (FGF)-2, FGF-7, FGF-10, and epidermal growth factor (EGF) [3]. Among them, laminin-111 has been reported to impair cellular response to surface stiffness and nuclear localization of YAP [27], indicating that its deposition on PAN-C nanofibers may be crucial for enhancing biocompatibility (Fig. 5A). In addition, laminin-111 and FGF-2 are also known to synergistically induce acinar differentiation of epithelial progenitor cells [28], potentially enhancing the yield of functional acinar cells in in vitro-cultured organoids.

To assess the adsorption of catechol moieties present on the PAN-C surface, we measured the WCA of PC, PAN film, and PAN-C film before and after albumin treatment. Albumin, being a highly hydrophilic protein, causes a decrease in WCA on albumin-adsorbed substrates. PC and PAN film showed no significant change in WCA before and after albumin treatment, indicating no albumin adsorption on the surfaces (Fig. 5B and C). However, after albumin treatment, PAN-C film exhibited a significant decrease in WCA, dropping from 56.0 ± 3.2° to 48.0 ± 1.7° (*P* = 0.0322). These results showed the adsorption of albumin molecules onto the PAN-C surface (Fig. 5B and C). Subsequently, the protein binding capacities of PAN and PAN-C nanofiber scaffolds were evaluated using FITC-labeled albumin. After immersing PC, PAN nanofiber, and PAN-C nanofiber in FITC-labeled albumin, they were thoroughly washed and subjected to fluorescence measurement. The mean value of FITC intensity of albumin-treated PAN-C nanofiber was approximately 430- and 3-fold higher than that of PC and PAN nanofiber, respectively (*P* < 0.001), demonstrating effective protein adsorption on the PAN-C nanofiber scaffold (Fig. 5D and E). Finally, adsorption of purified laminin-111 and FGF-2 on PC, PAN nanofiber, and PAN-C nanofiber was investigated. Consistent with the albumin adsorption assay results, PAN-C showed a significantly higher adsorption rate for laminin-111 and FGF-2 than that of PC (*P* < 0.001) or PAN (*P* < 0.001) (Fig. 5F and G).

### Enhanced functional differentiation in eSG organoids cultured on PAN-C

**Fig. 6.**
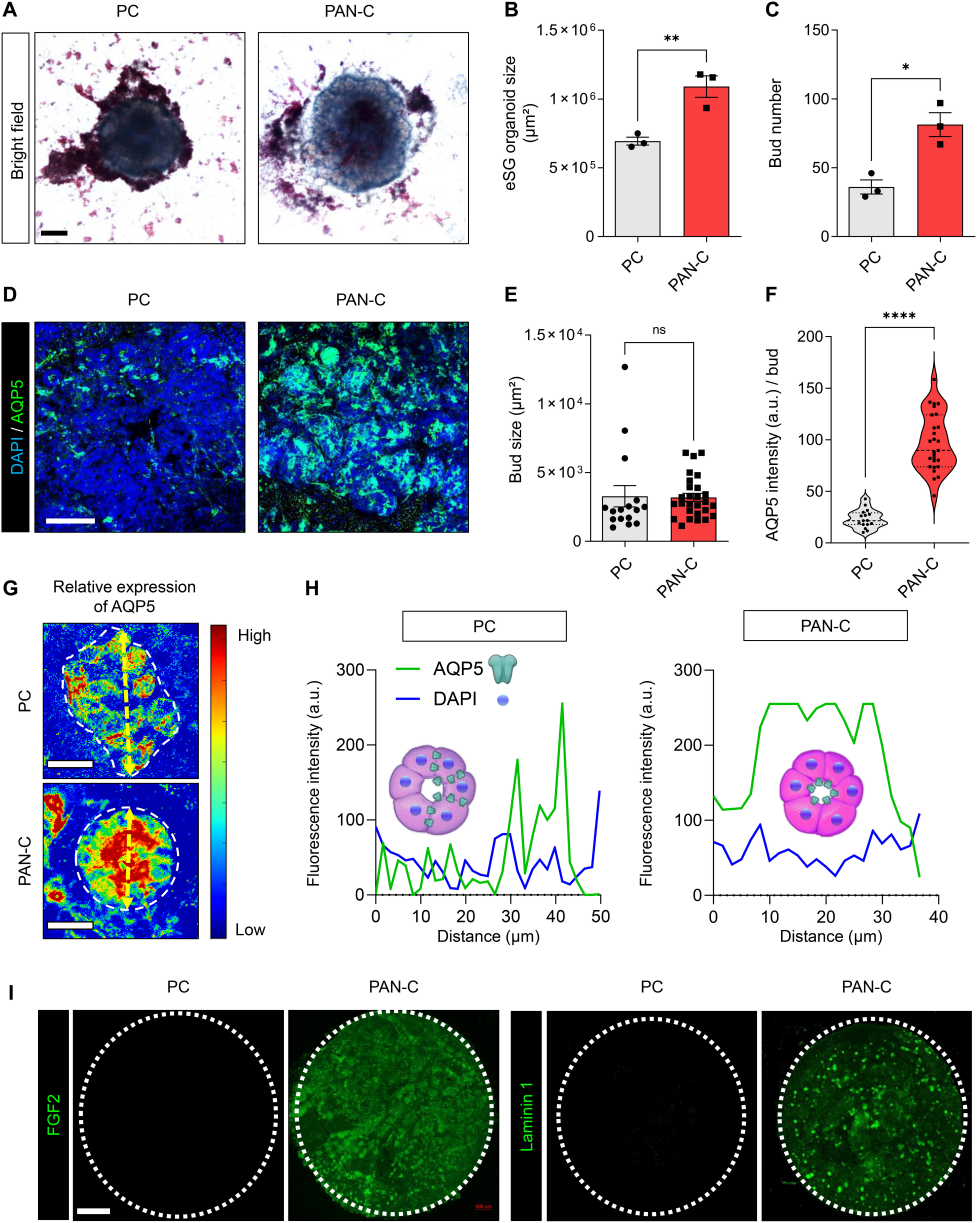
Morphogenesis and functional differentiation of eSG organoids cultured on PC, PAN nanofiber, and PAN-C nanofiber. (A) Bright-field images of eSG organoids on PC and PAN-C. Scale bar = 200 μm. (B and C) Quantification of eSG organoid size and bud number (*n* = 3 to 16). (D) Confocal images of eSG organoids stained with DAPI (blue) and aquaporin 5 (AQP5) (green). Scale bar = 100 μm. (E and F) Quantification of bud size and AQP5 fluorescence intensity per bud (*n* = 3 to 16). (G) Heatmap depicting relative AQP5 fluorescence intensity in buds of eSG organoids. Each bud of organoids is marked with a white dotted line. The yellow double-headed arrows indicate the longest crossline through the center. Scale bar = 20 μm. (H) Quantification of AQP5 and DAPI intensity along with distance indicated by the yellow double-headed arrows in (G). (I) Confocal images of PC and PAN-C immunostained with fibroblast growth factor 2 (FGF-2) and laminin 1 after organoids removal (representative of triplicates). The white dotted circles indicate the boundaries of each circular PC and PAN-C membrane. Scale bar = 1 mm. Statistical significance is set as *P < 0.05, **P < 0.01, ****P < 0.0001; ns, not statistically significant.

Salivary gland organoids can be established using either endogenous tissue-resident stem/progenitor cells [29] or eSG stem/progenitor cells [13,28], which can be derived from induced pluripotent stem cells (iPSCs). However, achieving a high yield of functional acinar cells in an in vitro culture system is challenging in both cases without supplementing exogenous laminin-111 or FGF-2, which limits scalability and economic feasibility [28]. Based on the strong protein adsorption capacity and biocompatibility of PAN-C, we hypothesized that the spontaneous deposition of laminin-111 and FGF-2 could enhance acinar differentiation in eSG organoids without additional supplements. To test this hypothesis, embryonic day 15 eSMGs, 1.5 days before acinar differentiation, were dissociated into single cells and re-seeded on PC membrane or PAN-C nanofiber scaffold. Re-seeded dissociated eSMG cells can self-organize into spherical organ germs or organoids [30]. The eSG organoids cultured on a PAN-C nanofiber scaffold exhibited 1.5 times larger organoid size (*P* = 0.0088) and 2.3 times higher bud number (*P* = 0.0109), indicating that PAN-C promoted the proliferation of organoids (Fig. 6A to C). Specifically, the average projected area of a single organoid cultured on PAN-C scaffolds for 48 h was approximately 1,090,401 μm^2^ (0.0109 cm^2^), compared to 693,589 μm^2^ (0.0069 cm^2^) on PC membranes. To further clarify whether the limited adsorption of laminin-111 and FGF-2 on PAN nanofibers contributes to organoid development, eSG organoids were cultured on PAN nanofiber scaffolds. In eSG organoids cultured on PAN, self-organization into spherical organoid structures was not observed, and AQP5 expression was markedly lower than in organoids cultured on PC (Fig. S6). These results suggest that, although PAN nanofibers exhibit measurable adsorption of ECM proteins and growth factors (Fig. 5F), the extent of biomolecular deposition is insufficient to overcome the negative effects of high substrate stiffness. Next, to further investigate bud size and acinar differentiation of eSG organoids, DAPI and AQP5 were visualized using immunofluorescence. While PAN-C did not affect the bud size of eSG organoids (*P* = 0.9048) (Fig. 6D and E), it significantly increased AQP5 expression in the buds (*P* < 0.0001), thereby enhancing acinar differentiation (Fig. 6D and F). To ensure proper saliva production, acinar cells must maintain their polarity, localizing the expression of AQP5 to the apical side of the cells [31]. Epithelial buds expressing AQP5 in eSG organoids cultured on PC displayed disrupted polarity, resulting in irregular spatial expression patterns of AQP5 (Fig. 6G and H). However, in the PAN-C nanofiber group, AQP5-expressing epithelial buds maintained polarized expression solely at the apical side (lumen side) of acinar cells (Fig. 6G and H). To confirm that this effect resulted from the spontaneous accumulation of FGF-2 and laminin-111 on PAN-C nanofiber, immunofluorescence staining for FGF-2 and laminin-111 was performed after detaching eSG organoids from PC membrane or PAN-C nanofiber scaffolds. PAN-C showed significant deposition of FGF-2 and laminin-111 than those observed on the PC membrane (Fig. 6I). These results showed that PAN-C nanofiber scaffolds can serve as a reservoir for ECM and growth factors produced by mesenchyme, thereby extending their bioactivity and substrate-masking effects even after the mesenchyme becomes inactive or perishes.

## Discussion

Effective embryonic organoid models are critical for progress in regenerative medicine and the tissue engineering of complex glandular structures, including the salivary gland. Previous studies have demonstrated the potential of embryonic organoids as transplantable bioengineered tissues [6,32]. For instance, Ogawa et al. [6] reported that bioengineered salivary gland organ germs successfully replaced dysfunctional salivary glands and restored secretory function upon transplantation. Building on such foundational studies, our approach integrates the developmental principles underlying organ germ formation with an embryonic niche-mimicking platform to generate structured glandular tissues that could serve as functional replacements for damaged glands.

While embryonic tissue-derived organ germs are not clinically feasible, recent advancements in stem cell biology and guided differentiation protocols offer promising alternatives by enabling the generation of eSG epithelial and mesenchymal components from iPSCs. Tanaka et al. [33] demonstrated the induction of primitive oral ectoderm from human iPSCs using bone morphogenetic protein 4 and a transforming growth factor-β inhibitor. Culturing this primitive oral ectoderm for 80 days in the presence of FGF-7 and FGF-10, growth factors naturally produced by eSG cells, successfully induced differentiation into salivary gland organoids expressing AQP5 [33]. These results underscore that chemical signals derived from eSG tissues can effectively guide in vitro modeling for generating artificial salivary glands.

Given that the ultimate goal of regenerative medicine is to replace lost or dysfunctional tissue with bioengineered alternatives, the next critical step is the systematic optimization of cellular composition, scaffold properties, and biochemical signals to maximize tissue functionality. Numerous studies have employed eSG organoid models to identify factors that enhance proliferation of eSG organoids and acinar differentiation. To achieve effective proliferation of eSG organoids, it is crucial to overcome the inherent challenge of their reduced growth rate in ex vivo cultures compared to natural in utero development, primarily due to insufficient support from niche factors [2,34]. To address this issue, previous studies have explored various biomaterials designed to mimic these niche conditions, including chitosan [35], hyaluronic acid [12–14,36], laminin [27,28], and amniotic membrane [37]. For functional differentiation of eSG organoids, FGF-2, laminin-111, and Rho-associated coiled-coil-containing protein kinase (ROCK) inhibitors have been shown to promote acinar differentiation [28,38]. Additionally, it has been observed that platelet-derived growth factor receptor alpha (PDGFRα)-expressing adult stromal cells effectively facilitate proacinar cell differentiation in eSG organoids through FGF-2 signaling [39].

However, these approaches rely on costly and easily degradable growth factors and ECM components such as Matrigel, which limits their scalability and clinical applicability. Therefore, there is a pressing need to develop mechanically stable, long-lasting tissue engineering scaffolds capable of preserving growth factors and ECM components, thus enabling in vitro cellular proliferation and functional differentiation comparable to physiological conditions observed in utero. Our PAN-C nanofiber scaffold exhibits an exceptional ability to capture soluble growth factors and ECM proteins secreted by eSG cells, thereby maintaining a high local concentration of these factors specifically around the organoid-contact area. This property significantly enhances in vitro proliferation and acinar differentiation in eSG organoids.

Ensuring high mechanical stability and robustness in salivary gland tissue engineering scaffolds remains a major challenge, as excessively stiff substrates can disrupt epithelial branching morphogenesis, hinder epithelial cell proliferation, and impair functional differentiation [11,36]. In this study, this phenomenon was demonstrated as eSMGs cultured on unmodified PAN with high stiffness exhibited significantly increased YAP/TAZ associated gene such as Cyr61 [40] (Fig. 4C and D), along with decreased expression of epithelial stem/progenitor markers such as Sox9 [26,41,42] and Kit [43] and their proliferation indicators, Ccnd1 and Mcm2 (Fig. 4H). Attempts to address these challenges using softer, biomimetic materials, such as Matrigel [29] or decellularized amniotic membranes [37], have had limited success due to high cost, complex preparation methods, and insufficient mechanical robustness for long-term culture. Similarly, electrospun scaffolds composed of polycaprolactone (PCL) [44] or poly(lactic-co-glycolic acid) (PLGA) [45] exhibit tunable topologies but suffer from inherent stiffness.

Another important limitation of the previously developed biomaterials is their biodegradability. While biodegradability is often considered advantageous in tissue engineering, it becomes a drawback when scaffolds are used solely as platforms for in vitro organoid culture. In such cases, degradable materials pose a risk as their physicochemical properties gradually change [46], releasing by-products that disrupt the culture environment [47], compromise scaffold integrity, and hinder the reproducible production of organoids over time.

In this study, we demonstrate that catechol-incorporated PAN-C scaffolds overcome these limitations by enabling the spontaneous adsorption and prolonged retention of mesenchyme-derived factors. The unique properties of PAN-C scaffolds provide a stable, cost-effective platform that maintains long-term structural integrity while mitigating stiffness-induced limitations. Despite exhibiting comparable modulus values to unmodified PAN (Fig. 2F), PAN-C scaffolds reduced the expression of mechanical stress-related genes, such as Actg2 and Cyr61 (Fig. 4C), while restoring down-regulated epithelial progenitor markers including Sox9 and Kit.

To better frame the functional relevance of these transcriptional changes, we note that Cyr61 and Actg2 are mechanically responsive genes [22,24] whose suppression on PAN-C scaffolds reflects reduced matrix-induced tension and a more permissive biomechanical environment. In parallel, restoration of Sox9 and Kit expression suggests reactivation of epithelial progenitor identity and mesenchymal signaling—both prerequisites for branching morphogenesis [2,26,41]. Moreover, the apical localization of Aqp5 indicates reestablished epithelial polarity consistent with fluid transport readiness [31]. Together, these transcriptional shifts underscore how the PAN-C scaffold modulates the mechanical and biochemical niche to reinstate developmental programs essential for functional acinar organization.

Notably, the restoration of normal Cyr61 expression level—a key downstream target of YAP nuclear localization—suggests that PAN-C somehow masked the cell’s response to substrate stiffness. Kechagia et al. [27] reported that laminin-111 coating, unlike fibronectin or collagen I, prevents YAP nuclear localization in breast epithelial cells. Thus, we propose that cell-secreted laminin-111 deposition on PAN-C played a crucial role in preserving branching morphogenesis on stiff substrates. Another possible mechanism is that PAN-C’s catechol moieties may have directly inhibited YAP/TAZ activation. Haak et al. [48] demonstrated that dopamine or dopamine receptor D1 agonists suppress YAP/TAZ in lung fibroblasts, effective enough to reverse fibrosis. Additionally, our previous study showed that coating stiff substrates with catechol-conjugated hyaluronic acid mitigated stiffness-induced inhibition of eSMG branching morphogenesis [36]. It seems that the incorporation of catechol moieties into biomaterials appears to offer specific advantages for salivary gland tissue engineering, and further investigation is required for such enhancement effects in multiple aspects.

Hydrophobicity is often considered a critical parameter in scaffold design for tissue engineering [49]; however, it appears to be less influential in tissue-level culture of salivary glands. For instance, the conventionally used PC membrane exhibits strong hydrophobicity but does not impede eSG organoid formation or branching morphogenesis in the organotypic culture of eSMGs. Similarly, in our study, incorporating catechol into PAN scaffolds did not significantly alter their hydrophobic characteristics (Fig. 2G and H). Instead, incorporated catechol significantly enhanced scaffold bioactivity through the stable, catechol-mediated adsorption of functional biomolecules (Fig. 6). These findings suggest that catechol-based surface modification strategies could be broadly applicable to other synthetic polymer nanofibers, such as PCL and PLGA, regardless of their intrinsic hydrophobic properties.

Although the PAN-C nanofiber scaffold developed in this study was primarily designed and validated for the in vitro culture of eSG organoids, it also holds considerable potential as an implantable scaffold for promoting in vivo salivary gland regeneration. Given the excellent capacity of PAN-C scaffolds to capture biomolecules, paracrine growth factors and ECM components secreted by human mesenchymal stem cells or other regenerative cell populations could be effectively immobilized onto PAN-C via a simple coculture method. The resulting bioactive PAN-C scaffold could then be directly implanted onto injured salivary glands to enhance their regenerative processes. Although our preliminary in vivo study confirmed the biocompatibility and absence of toxicity of PAN-C scaffolds upon implantation into mouse SMGs, future studies are required to thoroughly assess their therapeutic efficacy in salivary gland regeneration.

Finally, optimizing the synthesis process is critical for translating PAN-C scaffolds to clinical applications. Fine-tuning the concentration and ratio of the catechol monomer would significantly influence polymerization kinetics, mechanical robustness, and biological functionality of the resultant PAN-C scaffolds. Due to the inherent radical-scavenging properties of catechol moieties, careful optimization is necessary to achieve an ideal balance between radical stabilization during polymerization and maintaining the desired biological and physical properties. Although alternative fabrication techniques such as 3-dimensional printing have attracted attention for their design flexibility, current limitations regarding speed, throughput, and cost hinder their scalability for mass production [50]. In contrast, electrospinning is already widely employed industrially through multi-nozzle systems, enabling continuous and large-scale production. Furthermore, the economic viability of PAN as a base material, commonly used in textile industries due to its low cost and high availability, positions PAN-C scaffolds as cost-effective solutions for tissue engineering. Integrating small quantities of catechol moieties into PAN scaffolds marginally increases overall material costs, yet substantially improves biological performance, aligning with the goals of creating advanced, economically feasible biomaterials for regenerative medicine.

## Conclusions

In conclusion, the catechol-incorporated PAN-C scaffold represents a transformative approach for salivary gland tissue engineering. By spontaneously capturing mesenchymal-derived ECM and growth factors, the scaffold establishes a microenvironment conducive to epithelial differentiation and functional maturation. These findings highlight the potential of catechol-incorporated biomaterials to serve as biologically active materials for eSG organoid culture and regenerative medicine applications, paving the way for scalable and clinically relevant tissue engineering solutions.

## Data Availability

The data are available from the corresponding authors on reasonable request.
