# Supplementary material for: Biomimetic Catechol-Incorporated Polyacrylonitrile Nanofiber Scaffolds for Tissue Engineering of Functional Salivary Glands
Source: Biomater Res. 2025 Jul 2;29:0226. doi: 10.34133/bmr.0226 (PMC12218223; doi:10.34133/bmr.0226)
Supplement: Supplementary 1 — Figs. S1 to S6 [file bmr.0226.f1.docx]

**Biomimetic Catechol-incorporated Polyacrylonitrile Nanofiber Scaffolds for Tissue Engineering of Functional Salivary Glands**

Seokjun Kwon^1†^, Ji Hyun Ryu^2†^, Junchul Kim^1†^, Hyun Ho Shin^2^, Gehoon Chung^1^, Ali Taghizadeh^3^, Jung-Hwan Lee^3^, Jongho Kim^4^, Bon-Cheol Ku^5^, Kyungpyo Park^1*^, and Sang-woo Lee^1,6*^

^1^Department of Physiology, School of Dentistry and Dental Research Institute, Seoul National University, Seoul, Republic of Korea.

^2^Department of Carbon Convergence Engineering, Department of Chemical Engineering, Smart Convergence Materials Analysis Center, Wonkwang University, Iksan, Jeonbuk, Republic of Korea.

^3^Institute of Tissue Regeneration Engineering (ITREN), Dankook University, Cheonan, Chungcheongnam-do, Republic of Korea.

^4^Department of Textile System Engineering, Kyungpook National University, Daegu, Republic of Korea.

^5^Institute of Advanced Composite Materials, Korea Institute of Science and Technology (KIST), Wanju, Republic of Korea.

^6^Center for Nanoparticle Research, Institute for Basic Science (IBS), Seoul, Republic of Korea.

*Address correspondence to: [kppark@snu.ac.kr](mailto:kppark@snu.ac.kr) (Kyungpyo Park); [goodman23@snu.ac.kr](mailto:goodman23@snu.ac.kr) (Sang-woo Lee)

†These authors contributed equally to this work


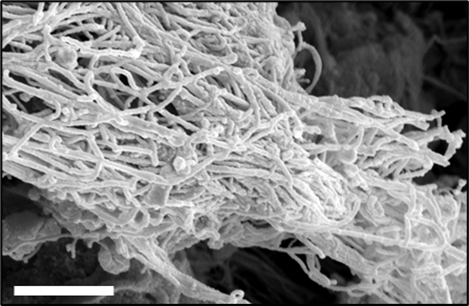


**Fig. S1.** SEM image. The mesenchyme of embryonic day 13.5 salivary glands consists of fibrous structures. Scale bar = 2µm.


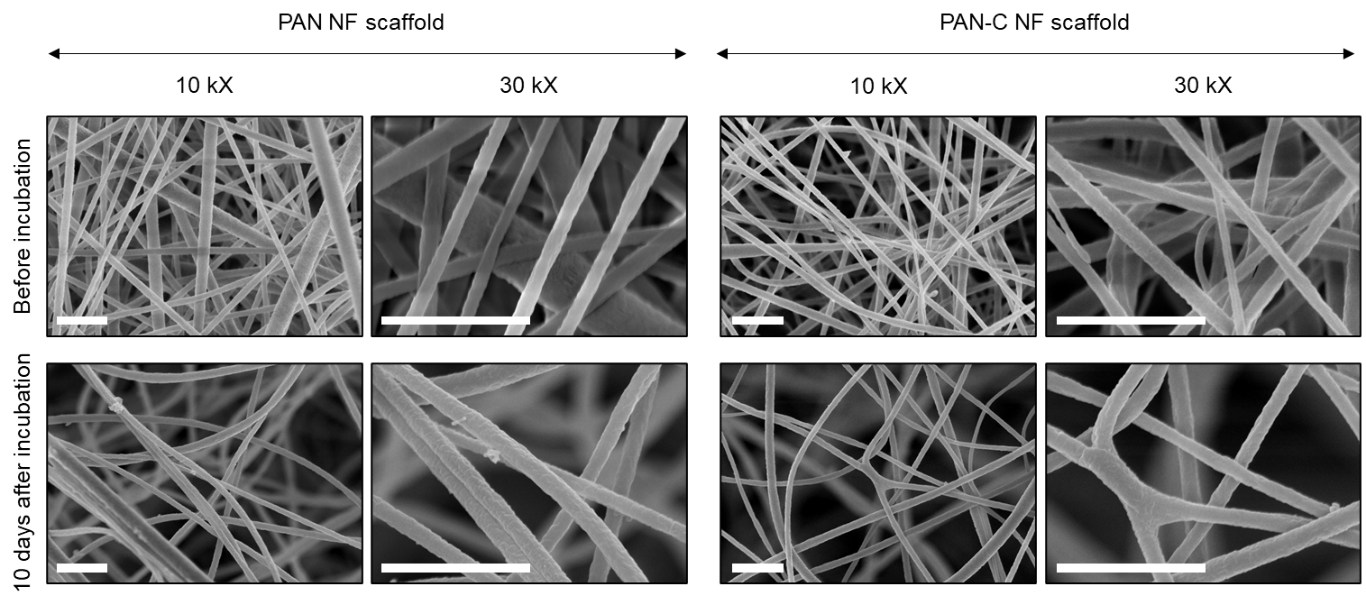


**Fig. S2.** SEM images of PAN and PAN-C nanofiber scaffolds before and after 10 days of DMEM incubation. Scale bar = 20µm.


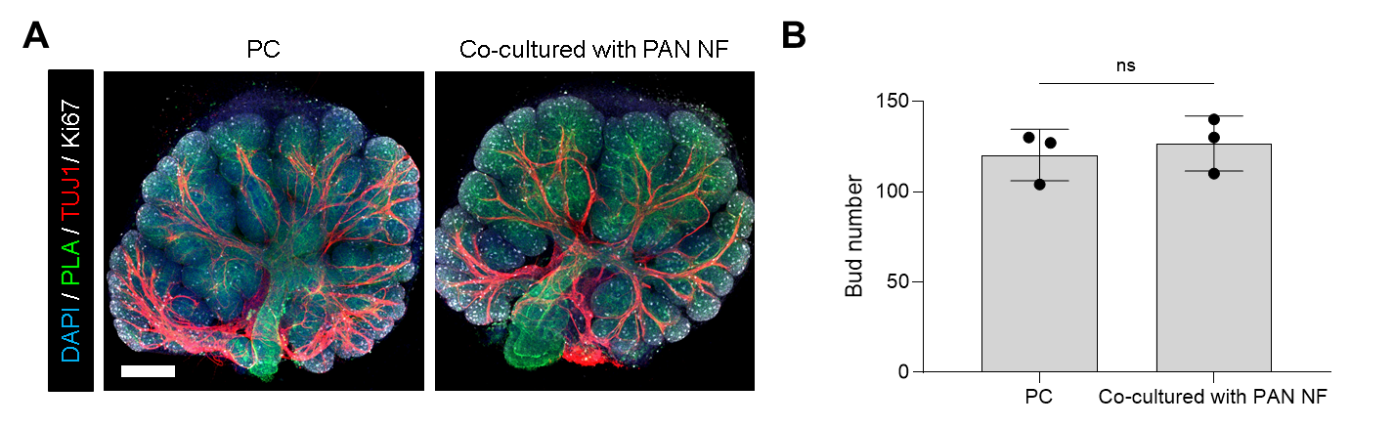


**Fig. S3.** Dissolution cytotoxicity test of PAN NF scaffolds on eSMG. (A) Representative immunofluorescence images of eSMGs cultured on PC membrane without/with presence of PAN NF scaffolds in culture system. Samples were stained with DAPI (blue), Ki-67 (white), PLA (green), and TUJ1 (red). Scale bar = 200 µm. (B) Quantification of bud number from the (A). Statistical significance is indicated as ns, not statistically significant.


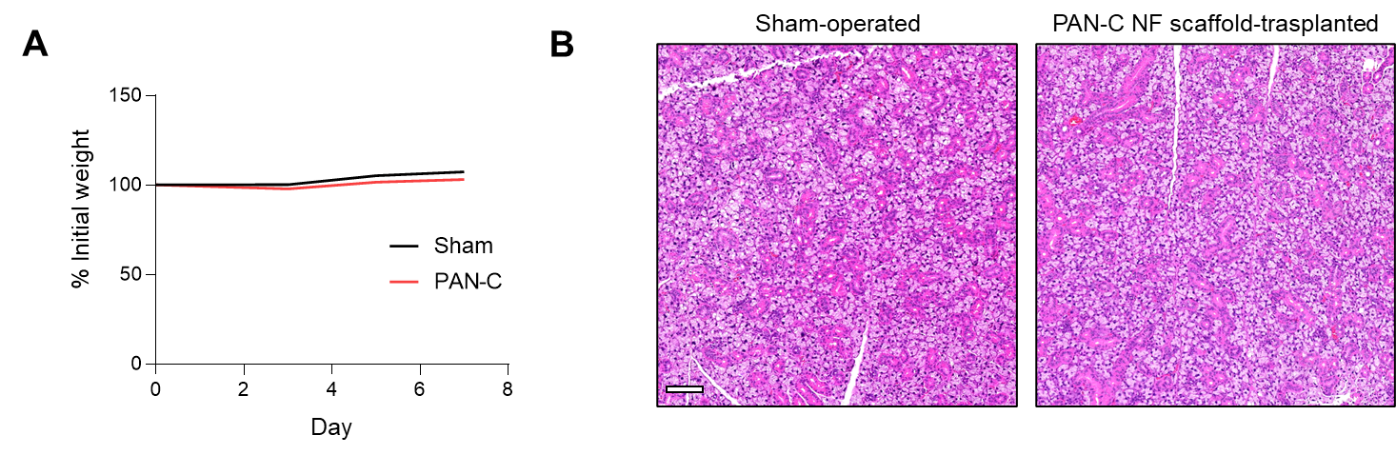


**Fig. S4.** *In vivo* biocompatibility of PAN-C nanofiber scaffolds. (A) Body weight changes of mice over a 7-day period following surgical implantation of PAN-C onto the submandibular gland surface, compared to sham-operated group. (B) Representative hematoxylin and eosin stained images of submandibular gland tissues collected from control and PAN-C implanted mice. Scale bar = 100 µm.


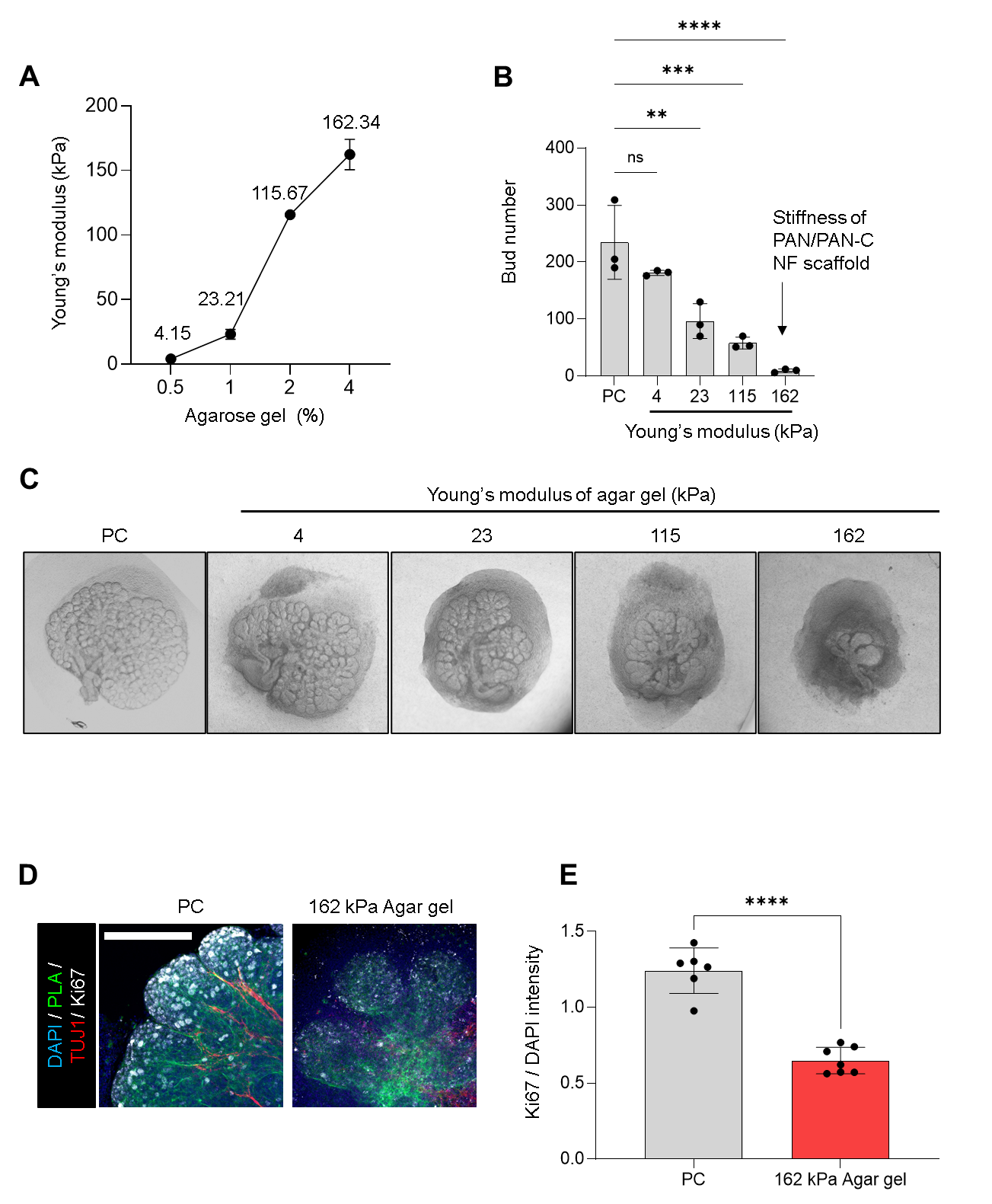


**Fig. S5.** Substrate stiffness-induced growth inhibition of eSMGs. (A) Young’s modulus of 0.5, 1, 2, and 4% agarose hydrogel measured by rheometer (n = 3). (B) Bud number of E13.5 eSMGs cultured on PC membrane and 0.5, 1, 2, 4% agarose hydrogels having Young’s modulus of 4, 23, 115, 162 kPa for 48 hours (n = 3). (C) Representative images of E13.5 eSMGs cultured on PC membrane and 0.5, 1, 2, 4% agarose hydrogels having Young’s modulus of 4, 23, 115, 162 kPa for 48 hours (n = 3). (D) eSMGs cultured on PC membrane and 4% agarose hydrogel having Young’s modulus of 162 kPa stained with DAPI (blue), PLA (green), TUJ1 (red), and Ki67 (white). Scale bar = 200 µm. (E) Quantification of Ki67 fluorescence intensity over DAPI (n = 6~7). Statistical significance is set as **P < 0.01, ***P<0.001, ****P < 0.0001; ns, not statistically significant.

**
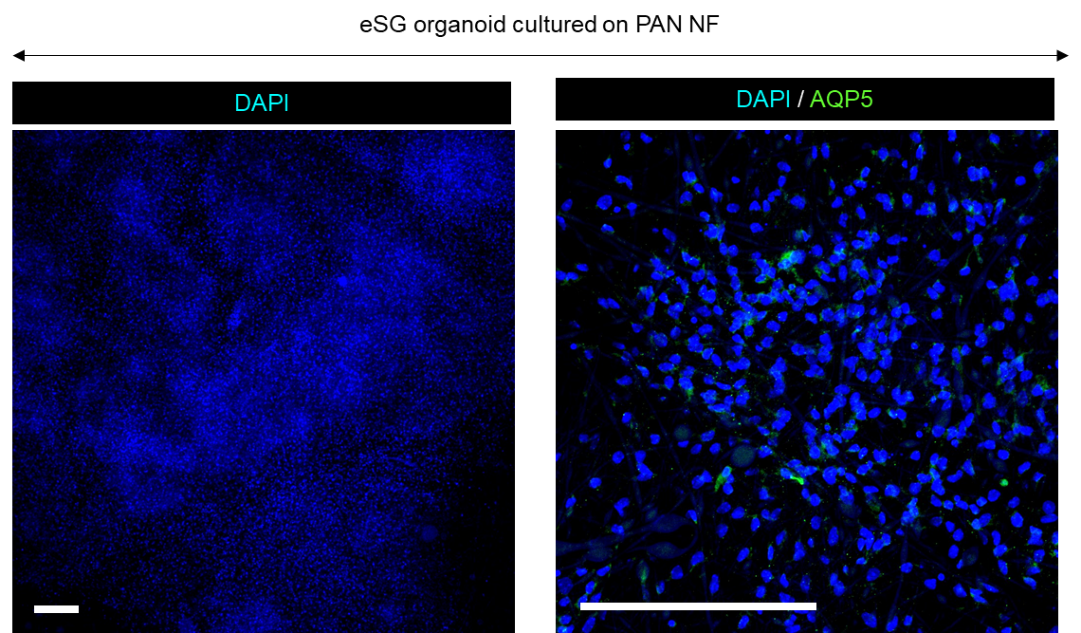
**

**Fig. S6.** Representative immunofluorescence images of eSG organoids cultured on PAN nanofiber scaffolds. The low-magnification view (left panel) shows DAPI staining, and the high-magnification view (right panel) shows DAPI (blue) and AQP5 (green) staining. bar = 200 µm.
